# Brown Tumors Due to Primary Hyperparathyroidism in a Patient with Parathyroid Carcinoma Mimicking Skeletal Metastases on ^18^F-FDG PET/CT

**DOI:** 10.3390/diagnostics5030290

**Published:** 2015-07-09

**Authors:** Kim Francis Andersen, Elisabeth Albrecht-Beste

**Affiliations:** Department of Clinical Physiology, Nuclear Medicine & PET, Rigshospitalet, Copenhagen University Hospital, Blegdamsvej 9—PET 3982, DK-2100 Copenhagen, Denmark; E-Mail: Elisabeth.Albrecht-Beste@regionh.dk

**Keywords:** brown tumors, parathyroid carcinoma, FDG PET, primary hyperparathyroidism

## Abstract

Parathyroid carcinoma only represents <1% of all cases of primary hyperparathyroidism (PHPT). Even rare, chronic PHPT may lead to excessive osteoclast activity, and the increased resorption leads to destruction of cortical bone and formation of fibrous cysts with deposits of hemosiderin—so-called brown tumors. These benign, osteolytic lesions may demonstrate FDG-avidity on ^18^F-FDG PET/CT, and as such are misinterpreted as skeletal metastases. Regression of the lesions may occur following successful treatment. We present a case demonstrating the diagnostic work-up and follow-up of a patient with PHPT due to parathyroid carcinoma and with presence of brown tumors on ^18^F-FDG PET/CT, visualizing the possible role of this imaging modality in the evaluation of treatment response in these patients.

**Figure 1:**
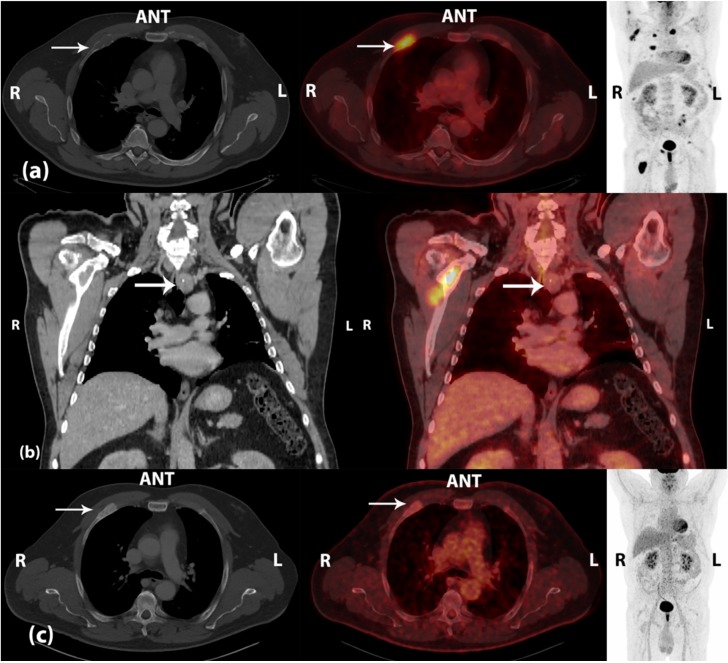
(**a**) Initial scan—left and middle: CT (bone window) and fused ^18^F-FDG PET/CT, transaxial view; right: ^18^F-FDG PET, maximal intensity projection. A 53-year old man with persistent bone pain localized to the knees, fatigue, and memory loss. Diagnostic work-up came out with strongly elevated plasma levels of parathyroid hormone (PTH, 180 pmol/L (normal range 1.18–8.43 pmol/L)) and ionized calcium (2.27 mmol/L (normal range 1.18–1.32 mmol/L)). Medical treatment with calcimimetics was initialized. The patient was referred to a fluorine-18 fluoro-2-deoxy-d-glucose positron emission tomography/computed tomography (^18^F-FDG PET/CT), which demonstrated multiple FDG-avid osteolytic and destructive lesions located to the peripheral and axial skeleton, some of the lesions with an extra-skeletal compartment. The lesions were suspected of being skeletal metastases. The white arrows point at a FDG-avid costal osteolytic lesion on the right side with maximal standardized uptake value (SUV_max_) of 7.2; (**b**) Initial scan—CT (mediastinal window) and fused ^18^F-FDG PET/CT, coronal view. In addition, a 2.5 cm paratracheal lesion with central calcifications located inferior to the left lobe of the thyroid gland was visualized on CT. The lesion demonstrated no pathological FDG uptake (SUV_max_ 2.8) and was concluded as being a parathyroid tumor (white arrows). Due to suspiciousness of primary hyperparathyroidism (PHPT), surgery was performed in terms of left-sided hemithyroidectomy and *en bloc* resection of the parathyroid tumor including regional lymph nodes. A spontaneous drop in PTH plasma levels of approximately 85% was seen 5 min after removal of the parathyroid tumor. Histopathology demonstrated parathyroid carcinoma with infiltrative growth pattern and invasion of vessels. The surgical margins were negative, and there were no lymph node metastases in the resected tissue; (**c**) Follow-up scan—left and middle: CT (bone window) and fused ^18^F-FDG PET/CT, transaxial view; right: ^18^F-FDG PET, maximal intensity projection. As staging of the disease was essential in terms of choice of treatment, a core needle biopsy from a FDG-avid osteolytic lesion in the left ilium seen on the pre-operative ^18^F-FDG PET/CT scan was performed. Histopathology was inconclusive due to lack of representative tissue. A follow-up ^18^F-FDG PET/CT scan was performed 3 months after surgery, and the previously seen osteolytic lesions were sclerotized with normal or only slightly increased FDG uptake. SUV_max_ of the previously mentioned costal lesion on the right side was now 2.5 (white arrows). In addition, both the patient’s symptoms as well as plasma levels of PTH and ionized calcium were normalized on further medical treatment. The lesions initially seen on ^18^F-FDG PET/CT were concluded as being benign brown tumors due to PHPT in this patient with parathyroid carcinoma. This case illustrates two clinical dilemmas: (1) A patient with parathyroid carcinoma, which was not visible on ^18^F-FDG PET due to lack of pathological FDG uptake in the tumor detected on CT; (2) The presence of multiple benign bone lesions with high FDG-avidity, which mimicked metastatic disease and as such could be misinterpreted. Parathyroid carcinoma is rare, representing <1% of all cases of PHPT [1]. Even though ^18^F-FDG PET/CT can offer information regarding lesion metabolism and potential loco-regional and/or distant spread of malignant disease [2], it probably has no place in the diagnostic work-up in a group of unselected patients with PHPT [3]. Despite the nomenclature, brown tumors are not true neoplasms, but rare, benign osteolytic lesions, which arise in the setting of excessive osteoclast activity in patients with hyperparathyroidism. The increased resorption leads to destruction of cortical bone and formation of fibrous cysts. Microscopically, hemosiderin deposition in the cysts gives a characteristic brown coloration and osteoclast-like giant cells may be present. The increased FDG uptake, which can be seen in these lesions (possibly due to the presence of osteoclast-like giant cells and macrophage glucose metabolism [4]), may mimic skeletal metastasis on ^18^F-FDG PET/CT. However, regression of the lesions has been reported after successful parathyroidectomy [5,6,7], supported by the findings in our case report. This indicates a possible role of ^18^F-FDG PET/CT in the evaluation of treatment response in these patients.
